# Evaluating a Large Language Model for Emergency Triage Using Synthetic Clinical Dialogues: Toward a Multimodal Clinical Safety Framework

**DOI:** 10.3390/diagnostics16203268

**Published:** 2026-10-09

**Authors:** Fatimah Albalawi, Reem Alotaibi, Sultanah Alshammari, Taha Masri

**Affiliations:** 1 Faculty of Computing and Information Technology, King Abdulaziz University, Jeddah 21589, Saudi Arabiasshammari@kau.edu.sa (S.A.); 2 Center for Research Excellence in Artificial Intelligence and Data Science, King Abdulaziz University, Jeddah 21589, Saudi Arabia; 3 Lifeline Research Unit, King Fahd Medical Research Center, King Abdulaziz University, Jeddah 21589, Saudi Arabia; 4 REGEDIM (Research Group on Emergency and Disaster Medicine), Vrije Universiteit Brussel (VUB), 1050 Brussels, Belgium; 5 Disaster Medicine Section, Emergency Medicine Department, King Abdulaziz University Hospital, Jeddah 21589, Saudi Arabia

**Keywords:** emergency department, triage, large language models (LLMs), Emergency Severity Index (ESI), decision-making, MIMIC-IV-ED, clinical safety, patient safety, Hajj healthcare, artificial intelligence in medicine

## Abstract

**Background/Objectives:** Prioritizing patient safety through accurate emergency triage is critical during mass-gathering events such as Hajj and Umrah, where high patient volumes can significantly increase the risk of clinical errors. Although the Emergency Severity Index (ESI) provides a standardized triage protocol, it remains dependent on human judgment, while traditional artificial intelligence (AI) models often struggle to capture the nuanced clinical context embedded in patient narratives. To address this gap, this study evaluates GPT-5.2 using synthetically generated dialogues as a step toward a conceptual clinical decision-support framework that bridges structured electronic health records (EHRs) with triage interactions through clinical dialogues. By combining narrative inputs with structured physiological indicators, it explores the potential to support more informed clinical decision-making under controlled simulation conditions. **Methods:** To evaluate this contribution, we systematically assessed the role of clinical dialogues in triage performance using a balanced and stratified dataset of 1000 simulated Hajj-context scenarios. **Results:** The results demonstrate that incorporating clinical dialogues can significantly improve triage performance, with a 79.7% relative reduction in under-triage across all ESI levels. A more noticeable improvement was observed in high-acuity cases, with a 91.9% relative reduction in under-triage. In addition, the model achieved a high-acuity recall of 94.5%, highlighting its ability to identify critical “red-flag” symptoms that are often not captured in structured data. These findings suggest that narrative context may contribute to more accurate triage assessment and improved identification of high-acuity cases. **Conclusions:** Consequently, when evaluated within a controlled decision-support setting, this study suggests that large language model (LLM)-based systems may improve triage reliability and may contribute to safer triage decision-making in high-demand clinical environments.

## 1. Introduction

Emergency Departments (EDs), also known as Emergency Rooms (ERs), serve as the frontline of patient care worldwide. These departments frequently operate under high pressure due to overcrowding, limited resources, and the complex requirements of prioritizing patients based on clinical urgency [1,2]. Overcrowding in EDs is recognized as a major global public health challenge [1,3], often resulting in treatment delays, an increased risk of medical errors, and a significant compromise to patient safety [4,5,6]. Efficient triage is therefore essential to ensure fair, timely, and effective patient care in these high-demand environments [6,7,8]. The triage process is a core function of EDs, designed to prioritize patients according to the urgency of their conditions [9]. Traditional triage relies heavily on human judgment, which can vary between clinicians and shifts, potentially leading to either delayed care for critically ill patients (under-triage) or unnecessary allocation of resources for less urgent cases (over-triage) [7].

These challenges are further amplified during mass-gathering events such as Hajj and Umrah, and particularly during Hajj, where millions of pilgrims converge for several days of religious rituals. The continuous movement of pilgrimage across different sites increases the risk of medical emergencies. According to Saudi Arabia’s Vision 2030 projections, the number of pilgrims is expected to exceed 10 million, which will place significant pressure on healthcare services. The Saudi Ministry of Health addresses this demand through strategically located permanent and temporary healthcare facilities at key sites, including the Holy Mosque, Mina, and Arafat [8]. During Hajj, EDs handle exceptionally high patient volumes, with many patients presenting with urgent or life-threatening conditions. In such high-demand contexts, prioritizing patient safety through accurate triage is critical to saving lives and reducing waiting times [8,9].

Traditionally, several five-level triage systems have been adopted, such as ESI, Canadian Triage and Acuity Scale (CTAS), and Manchester Triage System (MTS), to standardize care and support clinical decision-making [10]. While these structured systems are designed to reduce variability in clinical judgment, they do not completely eliminate it, particularly under high-pressure conditions. Residual variability in triage decisions may still occur, which can lead to misallocation of resources and under-triage of high-acuity patients, potentially compromising patient safety [2,4]. To further enhance and complement existing structured triage systems, machine learning (ML) and deep learning (DL) approaches have increasingly been explored to support automated severity prediction in clinical settings using structured clinical data, such as vital signs and chief complaints [11]. Tree-based models, ensemble learning, and neural networks have demonstrated improved performance, while more advanced architectures, including hybrid and attention-based models, aim to capture clinical patterns from textual records [1,3,5,8,11,12,13,14,15]. While these models have demonstrated improved performance, a key limitation remains in their heavy reliance on structured variables. This restricts the models’ capacity to leverage the rich, unstructured information essential for reflecting real-world triage practices [16].

In recent years, LLMs have increasingly been used in clinical decision support systems. This is usually because of their strong ability to understand and process complex clinical language. A growing body of literature [2,6,16,17,18] suggests that these models can effectively interpret complex symptoms and clinical narratives to enable more context-aware assessments of patient conditions. Despite this promise, a fundamental constraint persists: most existing works remain limited by their reliance on structured data or static textual representations. This reliance fails to account for the nuanced narrative context that occurs naturally during real clinical interactions, where such conversational depth is vital for making accurate triage decisions and effectively mitigating clinical risks [16].

Although recent efforts [18] have attempted to process real-world conversations, their scope has been limited to non-critical cases (triage levels 3–5). Additionally, their evaluation did not consider structured vital signs, which may play an important role in accurate triage classification. Existing approaches may not fully capture the complexity and dynamic nature of emergency triage workflows in high-pressure mass-gathering environments, such as during the Hajj pilgrimage. This limitation is particularly important, as effective triage often depends on the timely integration of verbal cues and clinical indicators, which are not always adequately represented in current models.

To address this gap, we systematically evaluate GPT-5.2 using simulated text-based triage dialogues combined with structured clinical data. The experimental evaluation focuses exclusively on assessing the contribution of clinical dialogues to improving ESI assignment under simulated conditions, where structured physiological indicators and clinical dialogues are jointly used as input to the language model.

This study also proposes a novel multimodal framework, presented as a conceptual design for future development. The framework aims to bridge the gap between structured EHRs and the dynamic nature of real-world triage interactions. Within this conceptual framework, a clinical feedback mechanism is incorporated to support safer triage reasoning and may help reduce the risk of under-triage in high-acuity cases.

Our findings suggest the potential value of integrating structured clinical data with clinical dialogues to improve triage performance. Accordingly, the proposed framework may offer practical benefits in supporting more reliable and timely clinical decision-making in high-demand environments.

To the best of our knowledge, this work represents one of the early attempts to generate synthetic nurse–patient interactions adapted to mass-gathering emergency triage scenarios with Hajj-context characteristics.

The contributions of this study are as follows:

1. Adapted from a clinical dataset, this work generates synthetic, clinically grounded nurse–patient dialogues for emergency triage in mass-gathering settings with Hajj-context characteristics. The dialogue generation process leverages structured EHR data to support controlled and reproducible clinical simulation.
2. We propose a novel conceptual multimodal LLM-based triage framework that integrates structured clinical data with unstructured clinical dialogues to support ESI-based classification. The framework introduces a dual-layer clinical feedback mechanism designed to model the interaction between heterogeneous clinical inputs and support iterative refinement of model reasoning. This approach provides a structured foundation for future extensions of LLM-based triage systems toward more comprehensive multimodal clinical decision support.
3. We conduct a systematic evaluation of GPT-5.2 under simulated conditions to assess the impact of incorporating clinical dialogue information into structured clinical data for ESI classification.

## 2. Materials and Methods

**Figure 1:**
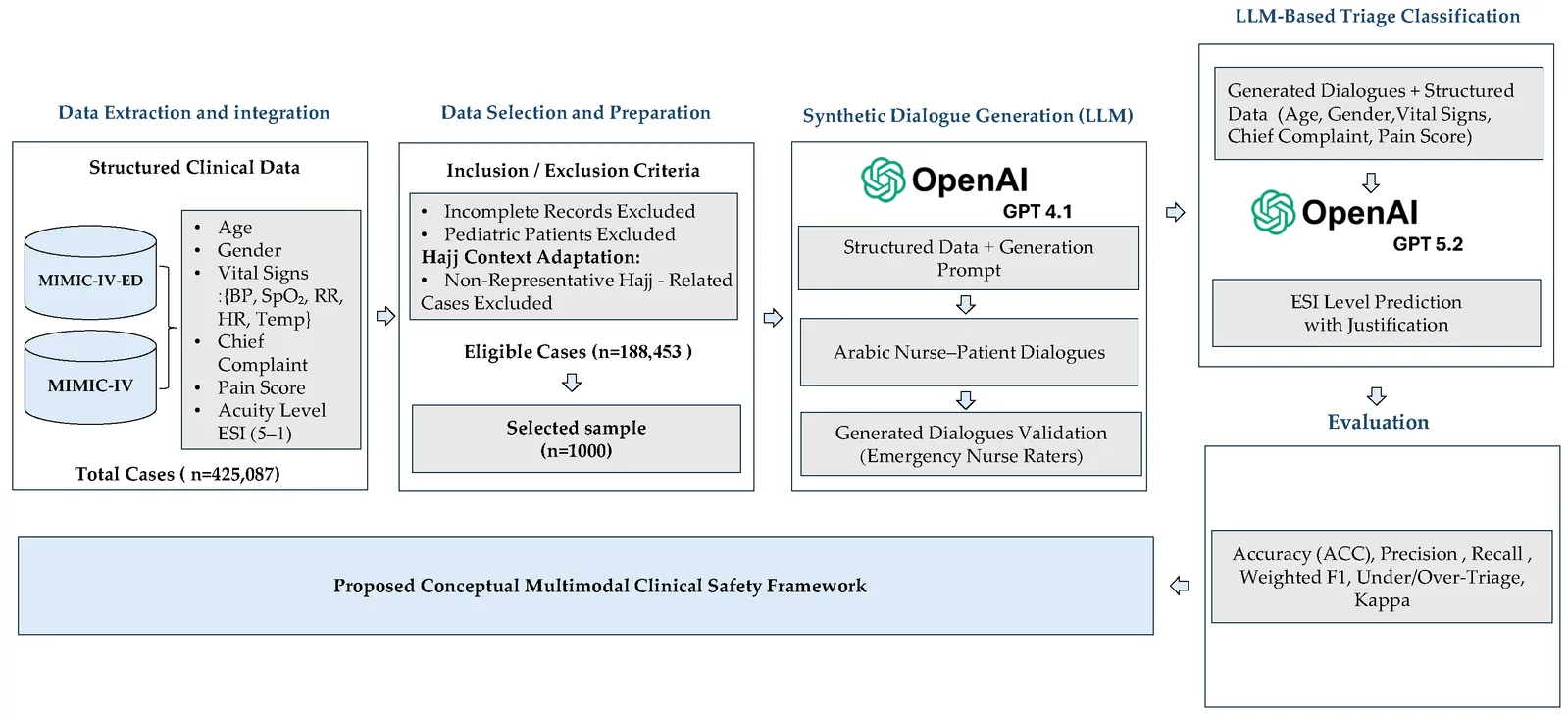
Overall study workflow.

In this study, we employed a retrospective study design to evaluate the performance of LLMs in emergency triage. Two large-scale, publicly available EHR databases were utilized: MIMIC-IV-ED v2.2 [19] and MIMIC-IV v3.1 [20]. Both databases include de-identified real-world clinical records and are maintained by the MIT Laboratory for Computational Physiology [21]. An overview of the study workflow, including data extraction and integration, case selection and preparation, synthetic dialogue generation and validation, ESI classification, and evaluation, is presented in Figure 1.

### 2.1. Data Acquisition and Processing

The MIMIC-IV-ED v2.2 database was used as the primary source of ED triage data in this study. It contains structured clinical variables that are aligned with the ESI protocol for 425,087 ED patient visits. These variables include vital signs such as heart rate, temperature, blood pressure, respiratory rate, oxygen saturation, systolic and diastolic, pain score, acuity level, and chief complaint. In addition, the database provides unique patient and visit identifiers, including subject_id and stay_id, which enable accurate tracking and analysis of individual patient encounters in the emergency department.

For the purpose of addressing missing demographic information, age and gender (anchor_age, gender) were extracted from the MIMIC-IV v3.1 database and integrated with the emergency encounters using the shared subject_id identifier.

All records are fully de-identified and were accessed in compliance with the official data use agreement to ensure patient privacy and adherence to ethical research standards. Table 1 provides an overview of all databases used, while Table 2 details the clinical variables extracted from each database for analysis.

### 2.2. MIMIC-IV-ED v2.2 Characteristics

The MIMIC-IV-ED database consists of 425,087 ED patient visits, representing a large and diverse real-world clinical population [19]. The demographic distribution of patients was relatively balanced, with approximately 54.10% females and 45.90% males.

Regarding age distribution, the majority of patients (73.64%) were adults, followed by elderly patients (25.33%), whereas pediatric cases accounted for a small proportion (1.03%).

A total of 58,362 distinct chief complaints were identified, which represents a wide range of clinical presentations. The triage acuity distribution is primarily composed of ESI level 3, followed by levels 2, 4, 1, and 5, which is consistent with typical ED triage patterns and supports the clinical realism of the dataset. Table 3 presents the gender distribution, Table 4 presents the distribution of age groups, and Table 5 presents the acuity distribution.

### 2.3. Case Inclusion and Exclusion Criteria

**Figure 2:**
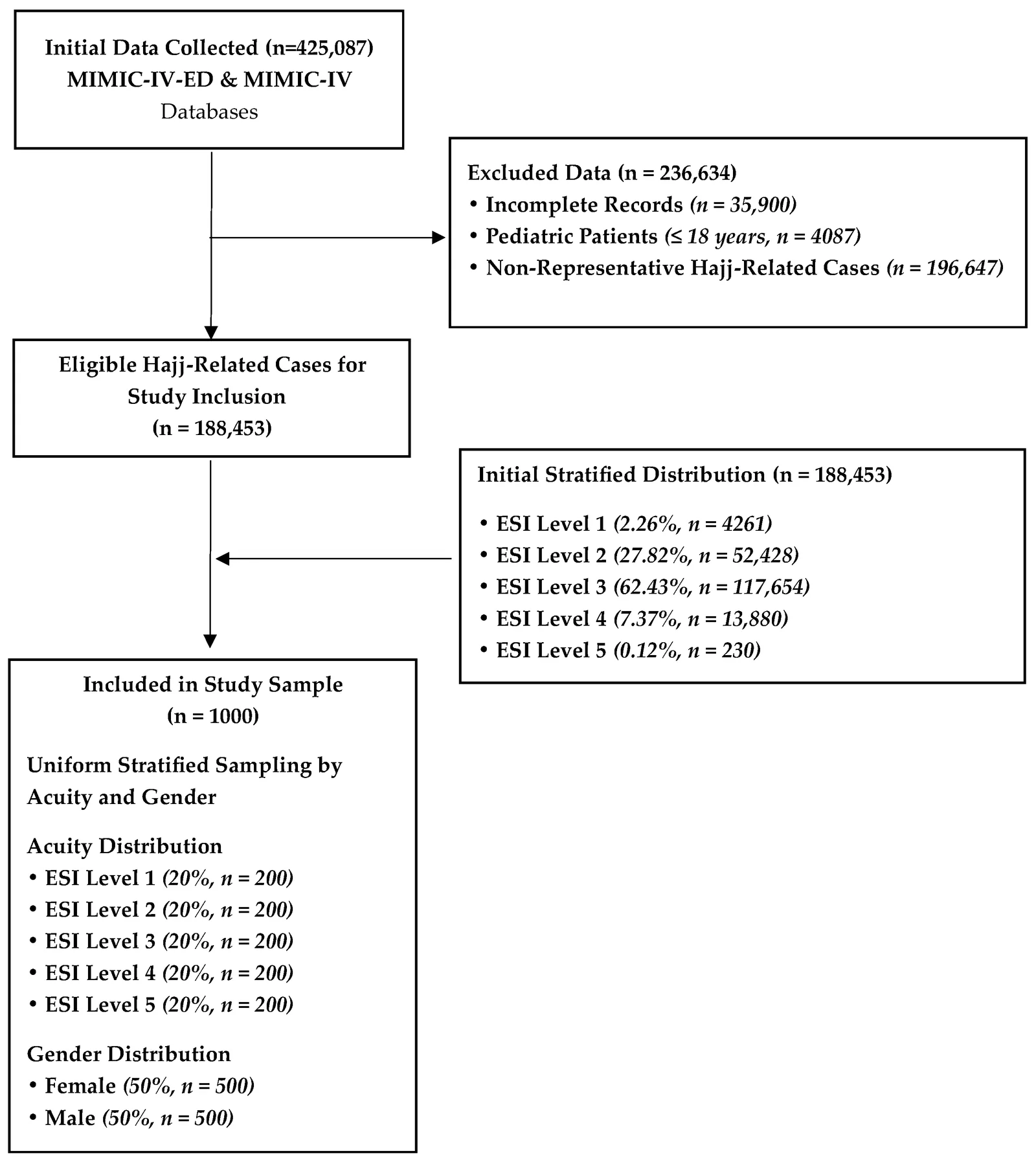
Data selection flow diagram.

In this study, we applied systematic inclusion and exclusion criteria to ensure data quality and to capture health conditions commonly associated with the Hajj. As mentioned earlier, the initial database consisted of 425,087 records. The data selection flow is illustrated in Figure 2.

In the first step, a total of 35,900 incomplete records were excluded due to missing essential vital signs or incomplete triage information. Then, to represent the demographic profile of Hajj pilgrims, the analysis was restricted to adult patients by excluding 4087 pediatric cases (aged ≤ 18 years).

Additionally, 196,647 records corresponding to clinical presentations that were not considered relevant or representative of the Hajj emergency-care context were excluded. The identification of Hajj-relevant clinical conditions was guided by a review of the relevant Hajj and emergency-care literature [22,23,24] to identify common and clinically relevant emergency presentations during Hajj. MIMIC-IV-ED records were then empirically examined, and cases with documented chief complaints compatible with these Hajj-relevant clinical presentations were retained, whereas cases classified as non-representative Hajj-related cases were excluded. This selection was performed as part of the Hajj-context adaptation of the dataset and was intended to construct a clinically appropriate cohort for Hajj-specific simulation and modeling, rather than to estimate the actual prevalence of emergency conditions during Hajj. The retained clinical conditions are summarized in Table 6. As a result, a final dataset of 188,453 eligible cases was obtained.

Finally, from this refined dataset, a subset of *n* = 1000 patients was selected using uniform stratified random sampling based on acuity level and gender. Specifically, 200 cases were sampled from each ESI level (1–5) to ensure balanced representation across severity levels. This sampling strategy provides balanced representation across acuity levels and patient demographics while preserving the diversity of clinical presentations present in the eligible cohort. Consequently, it supports a controlled evaluation of LLM-based triage performance.

### 2.4. Synthetic Dialogue Generation and Clinical Validation

To address the lack of recorded real-world conversations from the Hajj season, primarily due to privacy constraints and the logistical challenges associated with collecting authentic clinical dialogue data in such environments, we employed a systematic approach to generate synthetic clinical dialogues [25]. This process was not limited to simple text generation but instead constituted a structured clinical simulation designed to bridge the gap between static EHR data and dynamic emergency triage practices during the Hajj season. The Hajj context in this study is introduced solely as a simulation layer for controlled experimental evaluation and does not correspond to real-world Hajj patient data.

#### 2.4.1. Hajj Context Adaptation

The MIMIC-IV-ED database was adapted to the Hajj context by mapping patients’ chief complaints to conditions frequently encountered and common emergency health presentations reported during Hajj seasons [22,23,24]. In addition, clinical scenarios in the generation process were mapped to pilgrimage-specific activities such as Tawaf, Sa’i, Jamarat, prolonged walking, heat exposure, crowding, and physical exhaustion to capture the environmental and physical stressors associated with the Hajj experience. This adaptation serves as a clinically informed contextualization step for simulation purposes rather than a direct representation of real-world Hajj emergency encounters. Table 6 summarizes the included conditions used in the adaptation process.

Although structured data capture clinical measurements, such as vital signs and chief complaint, they may not fully reflect the decision-making process during triage, which is influenced by how patient information is communicated and interpreted. This motivates the incorporation of clinically grounded nurse–patient dialogues as an additional contextual component to investigate whether dialogue augmentation can improve ESI classification performance.

#### 2.4.2. Dialogue Generation and Prompt Design

To generate clinically meaningful and controlled nurse–patient triage dialogues, structured clinical information extracted from MIMIC databases, including demographic characteristics, vital signs, chief complaints, and predefined ESI labels, was incorporated into a carefully designed prompt template. Grounding the generation process in authentic emergency department data constrains the LLM to produce realistic dialogues that closely resemble real triage interactions while maintaining semantic and factual consistency with the underlying patient record. This structured prompt design also helps mitigate hallucinations and clinically inconsistent outputs by restricting the model to clinically relevant information and predefined patient attributes. Furthermore, the use of a standardized prompt template ensures a controlled, reproducible, and systematically evaluable dialogue generation process, enabling reliable assessment of LLM-based ESI classification [25].

This methodological design is further supported by recent studies demonstrating that grounding LLM-generated medical dialogues in structured clinical data improves clinical coherence and, when coupled with known ground-truth annotations, enables controlled and reproducible evaluation [26]. Compared with previous approaches that either focused on medical text generation without controlled ground-truth annotations [27] or relied primarily on static clinical texts [28], our approach builds upon similar methodological principles by integrating structured clinical grounding with standardized prompt engineering to generate realistic nurse–patient triage dialogues while preserving the underlying ground-truth clinical information for systematic evaluation of LLM-based ESI classification.

Simulated triage dialogues were generated using GPT-4.1 (OpenAI), which was prompted to act as an expert medical simulation agent. GPT-4.1 was selected for its strong natural language understanding and generation capabilities, enabling the production of contextually appropriate clinical conversations within the proposed dialogue generation framework [29].

**Figure 3:**
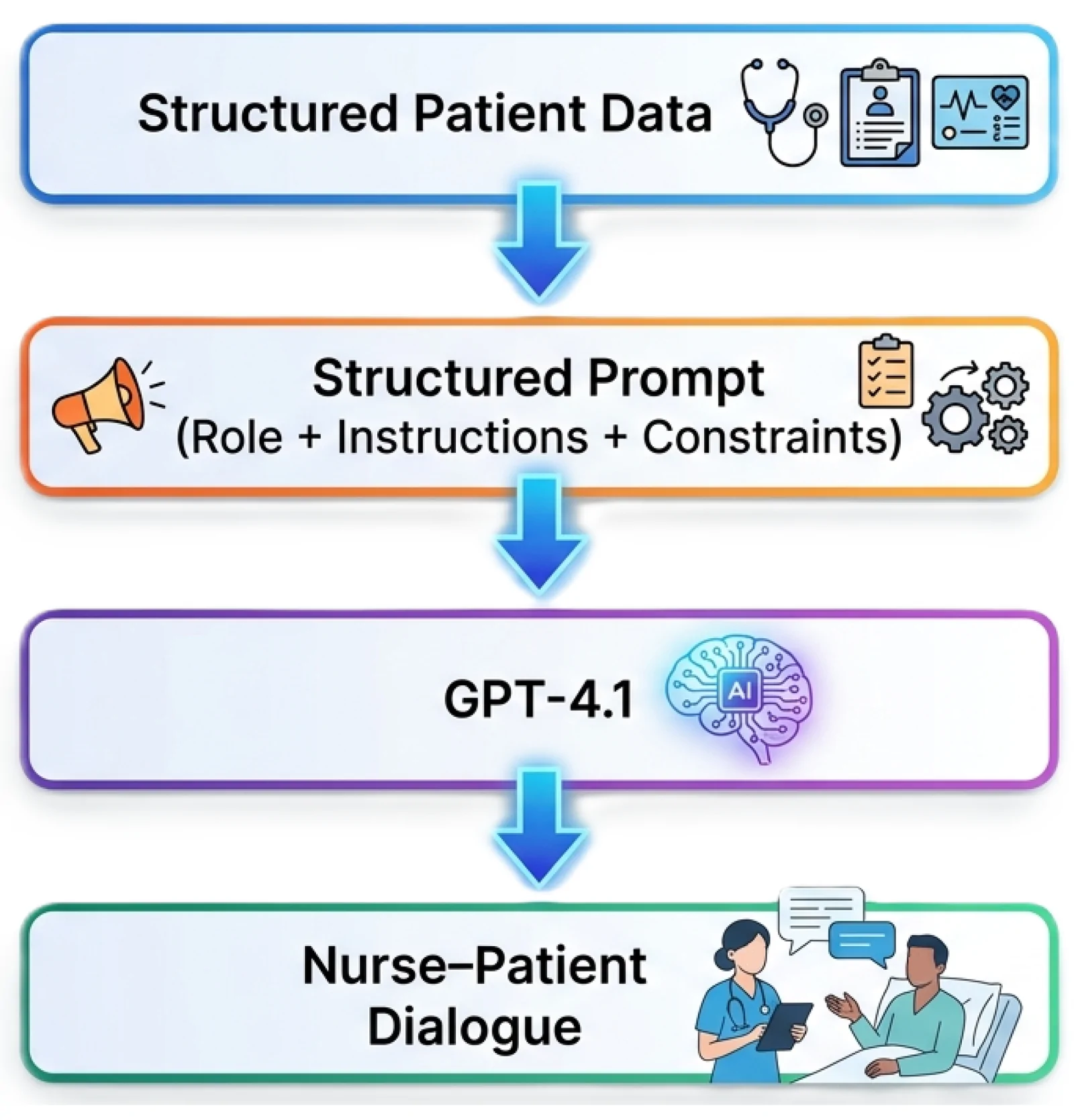
Overview of the nurse–patient triage dialogue generation process. Structured clinical data are integrated into a standardized prompt template to guide GPT-4.1 in generating clinically coherent and contextually appropriate nurse–patient triage dialogues.

To ensure the quality, consistency, and clinical relevance of the generated dialogues, a structured prompt template was developed. The prompt incorporated the patient’s structured clinical data, including demographic characteristics, vital signs, chief complaint, and predefined ESI level, together with explicit generation instructions to guide GPT-4.1 in producing realistic nurse–patient triage conversations. In addition, the prompt specified dialogue structure, clinical reasoning constraints, and output requirements to ensure semantic and factual consistency with the source patient record while minimizing the generation of unsupported or clinically inconsistent information. To achieve these objectives, the prompt was constructed according to a set of predefined design specifications that guided the dialogue generation process. These specifications were intended to standardize dialogue generation, improve reproducibility, and ensure that each generated conversation remained clinically coherent, contextually appropriate, and consistent with the corresponding patient record [25]. The general prompt specifications are summarized below. The overall nurse–patient triage dialogue generation process is illustrated in Figure 3.

**General Prompt Specifications:**

- **Role assignment:** GPT-4.1 was instructed to act as an expert medical simulation agent specialized in emergency triage.
- **Patient simulation:** Simulate realistic patient behavior, communication style, and responses that are consistent with the patient’s clinical condition, demographic characteristics, and assigned ESI level, ensuring clinically realistic nurse–patient interactions.
**Patient behavior across ESI levels:**
  1. ESI 1—Resuscitation (requires immediate life-saving intervention): Patient is critically ill, possibly unconscious or minimally responsive. Nurse interaction is brief and focused on airway, breathing, circulation, and consciousness. Patient may not respond, and information may be obtained from a bystander if needed.
  2. ESI 2—Emergent (high-risk situation): Patient shows signs of severe distress with limited responses. Nurse uses rapid, focused assessment to identify danger signs such as respiratory distress, neurological deficits, severe pain, or altered consciousness. Vital signs may appear deceptively stable.
  3. ESI 3—Urgent (requires two or more (≥2) internal resources): Patient is stable but requires further evaluation. Assessment focuses on symptom duration, severity, progression, functional impact, and associated warning signs.
  4. ESI 4—Less-Urgent (requires 1 internal resource): Patient is stable and requires limited evaluation. Dialogue reflects a straightforward assessment with expectation of a single intervention.
  5. ESI 5—Non-Urgent (requires 0 resource): Patient is fully stable and primarily seeks reassurance or advice. Interaction is minimal and focused on reassurance and basic guidance.
- **Clinical grounding:** Generate dialogues exclusively from the provided structured patient data.
- **Dialogue objective:** Produce realistic nurse–patient triage conversations that reflect authentic emergency department interactions.
- **Triage assessment:** Reflect the nurse’s systematic assessment process in accordance with standard ESI Version 4 triage practices [30].
- **Clinical consistency:** Ensure that all generated dialogue remains consistent with the patient’s demographic information, vital signs, chief complaint, pain score, and predefined ESI level.
- **Reasoning constraints and integrity:** The model must maintain clinical realism while preserving the implicit representation of the patient’s acuity, without explicitly revealing the ESI level, urgency, triage category, diagnosis, or treatment plan. Furthermore, it must not infer, fabricate, or introduce any clinical information (e.g., symptoms, diagnoses, laboratory findings, treatments, or management decisions) that is not relevant to the underlying clinical case.
- **Dialogue structure:** Follow a realistic emergency triage workflow using concise, natural, and clinically appropriate language.
- **Output format:** Adhere to the predefined dialogue structure and formatting requirements to ensure standardized and reproducible dialogue generation.

During the dialogue generation process, GPT-4.1 was instructed to follow a set of case-specific constraints designed to preserve clinical consistency, contextual relevance, and alignment with the corresponding patient record. These constraints ensured that each generated dialogue accurately reflected the patient’s clinical data while maintaining a realistic emergency triage interaction. For each individual case, the model was instructed to:

- Use the following patient information: age, gender, vital signs (temperature, heart rate, respiratory rate, oxygen saturation, systolic blood pressure, diastolic blood pressure), chief complaint, pain score, and predefined ESI level.
- Transform the patient information into concise, clinically realistic Arabic dialogues representing interactions between a triage nurse and a Hajj pilgrim.
- Do not generate any clinical information beyond what is explicitly supported by the patient record, including symptoms, diagnoses, laboratory findings, treatments, or medical management.
- Reflect the patient’s symptoms, functional limitations, and overall clinical presentation within the context of Hajj-related activities and environmental or physical stressors while preserving consistency with the original patient record.
- Maintain strict consistency with the provided patient information and predefined ESI level throughout the dialogue.
- Generate dialogues consisting of 3–5 turns, with shorter interactions for high-acuity cases (ESI 1–2) to reflect rapid triage assessment in time-constrained environments.
- Ensure that each dialogue includes clinically relevant observations, appropriate triage questions, and corresponding patient responses. Patient responses should use natural, everyday language (i.e., no medical jargon), while avoiding greetings, social conversations, or unnecessary narrative content to maintain a clinical focus.
- Follow the predefined dialogue structure and output format to ensure standardized and reproducible dialogue generation across all cases.

The generated dialogues constitute controlled and structured clinical simulations derived from EHR data. By preserving the underlying structured clinical information and predefined ground-truth ESI labels, the dialogue generation approach enables a standardized and reproducible evaluation of the contribution of dialogue augmentation to ESI classification under controlled experimental conditions.

### 2.5. Proposed Conceptual Multimodal Framework for Triage

This study proposes a conceptual multimodal LLM-based framework designed to enhance emergency clinical decision-making. The primary aim of this framework is to support a more comprehensive clinical representation by combining heterogeneous patient data for decision support in high-pressure emergency environments.

**Figure 4:**
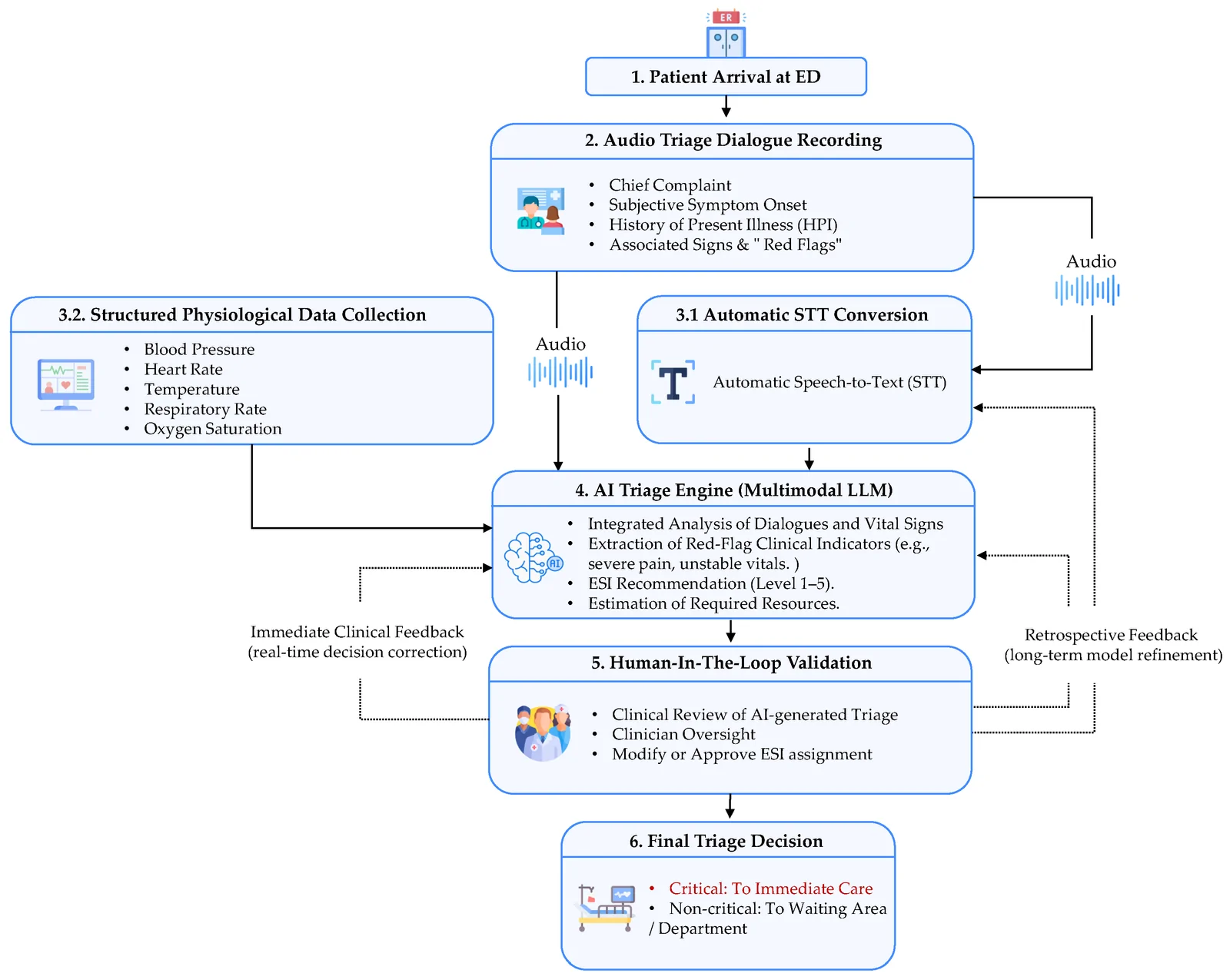
Overall architecture of the proposed conceptual multimodal LLM-based triage framework.

The experimental evaluation in this work is limited to text-based dialogues along with structured clinical data, where both structured physiological indicators and clinical dialogues are jointly provided as input to the language model. Furthermore, this study focuses on validating the model’s reasoning and ESI classification under simulated conditions, while full multimodal integration and clinical validation remain as future directions. Figure 4 illustrates the overall architecture of the proposed conceptual multimodal LLM-based triage framework.

Within the framework, structured clinical inputs include physiological measurements such as temperature, heart rate, blood pressure, respiratory rate, and oxygen saturation. Unstructured inputs are derived from triage dialogues, capturing key clinical information such as chief complaint, patient-reported symptoms, onset and progression, pain level, HPI, functional limitations, and potential red-flag indicators [30].

The framework follows a structured pipeline for multimodal integration:

1. **Data Acquisition:** Upon the patient’s arrival at the triage station, triage dialogues between nurses and patients are recorded via data acquisition tools, such as microphones integrated into the clinical workstation. At the same time, structured physiological data are simultaneously collected from connected medical devices.
2. **Data Integration and Centralized Processing:** Once the raw data is collected (audio recording and physiological data), it is transferred to a centralized system. Then, the resulting dialogue representations are integrated with the physiological data into a unified input format. This ensures that both narrative and numerical information are preserved for comprehensive analysis. Notably, the audio recordings can either be transcribed via a STT module or processed directly by a multimodal LLM for inference [18,31].
3. **LLM-based Inference:** In this step, the inputs are processed using LLM reasoning that follows ESI guidelines [30]. The model then extracts clinically relevant cues, identifies high-risk conditions, and generates an initial ESI-level classification as well as an estimation of required resources. The generated output is then transmitted to the nurse’s dashboard in real time. This interface provides a clear visualization of the reasoning behind the suggested triage level, which allows the nurse to review, validate, or override the decision directly at the point of care. This helps clinicians make timely and informed triage decisions while ensuring that the final decision remains under human oversight to support patient safety [6,31].

To facilitate continuous model refinement across heterogeneous data sources, this framework introduces a dual-layer feedback mechanism that consists of two complementary layers, described as follows:

- **Immediate Clinical Feedback (Human-in-the-Loop):** The triage nurse serves as an important safety layer within the framework. They provide human oversight of the LLM-generated recommendations. Once the model predicts the ESI level and clinical summary, clinicians can review, approve, modify, or override the output in real time. This interactive validation layer allows for the correction of potential AI hallucinations or data misinterpretations, which may help reduce the risk of misclassification. By enabling this clinician supervision, the human-in-the-loop approach aims to support safer clinical decision-making and has the potential to improve diagnostic reliability.
- **Retrospective Feedback and System Refinement:** This layer supports long-term system improvement by leveraging expert human corrections to iteratively refine both the STT engine and the Multimodal LLM’s reasoning behavior. By analyzing clinician modifications and overrides as retrospective feedback, the system aims to reduce performance discrepancies and improve the consistency of its clinical decision support over time. This creates a feedback-driven framework designed to learn from expert insights, thus supporting reliable decision support in demanding emergency environments.

### 2.6. Clinical Validation of the Methodology and Generated Dialogues

An emergency medicine expert reviewed the overall study methodology, including the dialogue generation process and the contextual adaptation of the clinical scenarios to the Hajj environment. The review confirmed that the proposed methodology was clinically appropriate and generally aligned with emergency triage practice, providing additional support for the clinical validity of the study design.

In addition, two experienced emergency nurses independently evaluated a random sample of 50 generated nurse–patient dialogues using a four-level rating scale to assess their clinical realism and relevance: Excellent (realistic and clinically relevant), Good (mostly realistic and clinically relevant with minor issues), Fair (somewhat realistic but with limited clinical relevance or coherence), and Poor (unrealistic or clinically irrelevant). Overall, the majority of the generated dialogues received Excellent or Good ratings, with only a few dialogues rated as Fair, indicating that the generated dialogues were generally perceived as clinically realistic and relevant.

These validation results supported the use of the generated dialogues in the subsequent experimental evaluation.

#### Comparison of Generated Dialogues with Structured Data

To assess the extent to which the generated dialogues provided contextual or narrative information beyond the corresponding structured records, we conducted a focused exploratory comparison of 50 randomly selected dialogues from the clinically validated sample. Each dialogue was reviewed against its corresponding structured patient record. Clinical information expressed in the dialogue was identified and classified as (1) directly represented in the structured data, (2) contextualized or rephrased information derived from the structured data, or (3) new clinical information not represented in the corresponding structured record. The proportions of information items in each category were calculated across the complete set of reviewed dialogues. In this exploratory comparison, approximately 35.95% of the identified clinical information items were directly represented in the structured data, while 64.05% were contextualized or rephrased representations of information already available in the structured records.

## 3. Experiments

All experiments were conducted in a simulation-based setting using Python (version 3.11.9) within the Visual Studio Code environment (version 1.109.0). The inputs consisted of simulated clinical dialogues in textual form, along with structured physiological data provided to the language model. GPT-4.1 and GPT-5.2 models were accessed and evaluated via the official OpenAI API (OpenAI, San Francisco, CA, USA) using the Python OpenAI library. GPT-5.2 was selected for ESI triage classification due to its advanced reasoning capabilities and its ability to effectively process structured clinical data. Several studies have reported strong performance of GPT-based models in complex reasoning and clinical language tasks (e.g., [2]), supporting its suitability for this study. In addition, GPT-5.2 had not yet been specifically evaluated for emergency triage tasks, which further motivated its selection in this study [32]. It is important to note that GPT-4.1 was used exclusively for dialogue generation, whereas GPT-5.2 was used for ESI classification. Maintaining separate models for these tasks ensured independence between the dialogue generation and triage classification stages, thereby enabling an objective assessment of patient acuity.

**Experimental Design:** Our primary objective was to investigate whether the inclusion of clinical triage dialogues could improve GPT-5.2’s classification performance. To explore this, we designed four controlled experiments to systematically evaluate how patient dialogue integration and prompt-engineered clinical reasoning influence model performance. Table 7 summarizes the experimental strategies, input data configurations, and prompting techniques used in this study.

Exp 1 and 2 employed standard zero-shot prompting, utilizing either structured clinical data alone or structured data augmented with generated triage dialogues. This setup served to quantify the direct contribution of clinical dialogues to the model’s triage decision-making performance. On the other hand, Exp 3 and 4 utilized R-CoT prompting. This approach incorporated clinical reasoning based on the ESI v4 protocol. The model was guided to reason step-by-step through the triage process while prioritizing critical clinical cues extracted from dialogues to refine urgency levels.

Furthermore, to ensure a controlled evaluation setting, all experiments were conducted within a fixed GPT-5.2 framework, where only the input configuration is systematically varied (structured data vs. structured data with clinical dialogues, and zero-shot vs. rule-constrained reasoning). The structured-data input consisted of the patient’s demographic characteristics (age and gender), chief complaint, vital signs (temperature, heart rate, respiratory rate, oxygen saturation, systolic blood pressure, and diastolic blood pressure), and pain score. This controlled evaluation design enables an isolated assessment of the impact of clinical dialogue on ESI triage performance under consistent model conditions as well as its interaction with structured reasoning prompts.

All reported results are based on a single experimental run under fixed prompts and configuration settings. No multimodal components were evaluated in this study. Furthermore, the full experimental pipeline, including prompts, model configurations, and representative outputs, is fully documented in the Supplementary Materials to ensure reproducibility.

**Evaluation Metrics and Statistical Analysis:** To systematically evaluate the model’s performance in emergency triage classification, a set of performance metrics and statistical analysis were employed. These metrics provide a comprehensive assessment of the model’s predictive performance and clinical reliability across all experimental conditions. The predictions were compared against ground-truth labels derived from nurse triage assessments.

The metrics used included overall accuracy, weighted F1-Score, precision, recall, under-triage rate, and over-triage rate. A confusion matrix was also used to show in detail how the model’s predictions were distributed across different ESI levels and to highlight common patterns across ESI levels. In addition, Weighted Cohen’s Kappa was calculated for Exp 1 and 2 using the full evaluation dataset (*n* = 1000) to further assess agreement with the ground-truth ESI labels and to compare agreement before and after incorporating the generated nurse–patient dialogues. To assess the reliability of these metrics, 95% confidence intervals were also calculated. To examine whether there were significant differences in triage classification performance across the evaluated strategies, Cochran’s Q test was applied. When significant differences were detected, pairwise post hoc comparisons were subsequently performed using McNemar’s test with Bonferroni adjustment to control for multiple comparisons. The primary performance metrics were computed for all four experiments, each conducted on a balanced dataset of 1000 cases to ensure uniform representation across all ESI levels.

For comparison purposes, multiple conventional supervised machine learning baselines, including Random Forest, Gradient Boosting, LightGBM, XGBoost, Logistic Regression, and Decision Tree, were implemented using the same structured clinical variables employed in Exp 1 (Structured Only). The dataset was partitioned using a stratified 80/20 train–test split to preserve the class distribution across the five ESI levels. All baseline models were evaluated on the same test set using the same evaluation metrics to provide a broader conventional machine learning reference for comparison.

## 4. Results

The final analysis included 1000 unique cases, each of which was evaluated across four experimental conditions. Table 8 summarizes the key performance metrics, including accuracy, precision, recall, F1-score, under-triage rate, and over-triage rate, as well as the average end-to-end response time from request submission to ESI-level assignment.

### 4.1. Impact of Clinical Dialogues on Triage Performance

Based on the results, Exp 2, which incorporates clinical dialogues, achieved the highest overall accuracy of 71.1% (95% CI: 68.3–73.9%), a precision of 75.9% (95% CI: 73.3–78.5%), a recall of 71.1% (95% CI: 68.3–73.9%) and an F1-score of 0.690 (95% CI: 0.66–0.72), compared to Exp 1, which achieved an accuracy of 41.8% (95% CI: 38.7–44.9%), a precision of 59.4% (95% CI: 56.4–62.5%), a recall of 41.8% (95% CI: 38.7–44.9%), and an F1-score of 0.366 (95% CI: 0.34–0.40).

**Figure 5:**
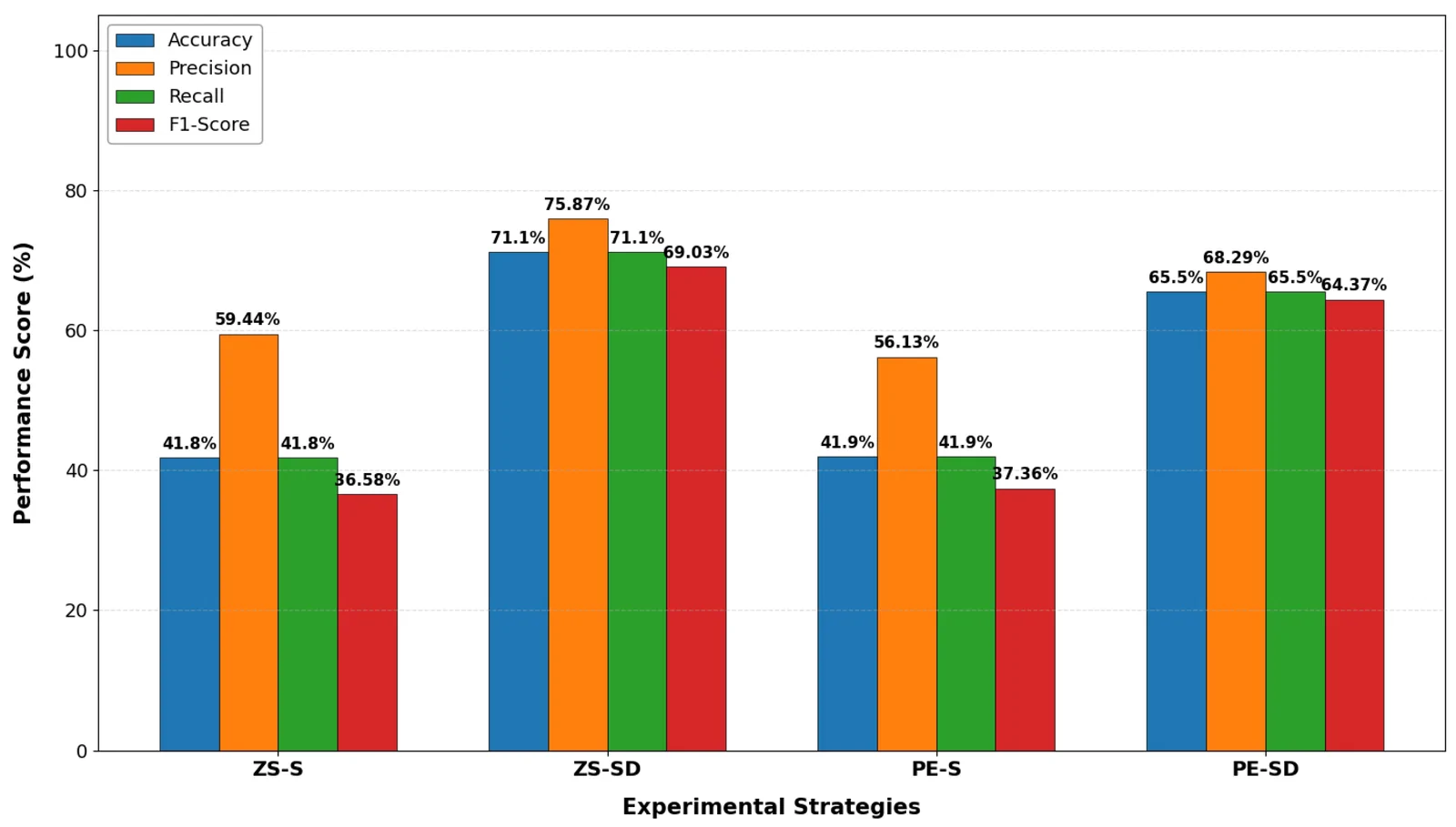
A comprehensive comparison of accuracy, precision, recall, and F1-score across the four experimental setups. For clarity, the abbreviations represent: ZS-S (Zero-Shot–Structured); ZS-SD (Zero-Shot–Structured & Dialogues); PE-S (Prompt-Engineered–Structured); and PE-SD (Prompt-Engineered–Structured & Dialogues).

This indicates a clear relative improvement in overall accuracy (+70.1% vs. Exp 1) and a notable reduction in under-triage from 31.6% to 6.4% (−79.7% vs. Exp 1). Additionally, in Exp 2, the improvement in recall highlights that the model became more effective in identifying urgent cases, which is especially important in triage scenarios where missed high-acuity patients may compromise patient safety. As shown in Table 8 and Figure 5, the inclusion of patient dialogues is associated with improved triage performance across all evaluation metrics. The table also highlights the changes in under-triage and over-triage rates. It shows a clear reduction in under-triage without a notable rise in over-triage. This suggests that the model may have potential implications for patient safety by more accurately identifying urgent cases while at the same time avoiding unnecessary pressure on clinical resources.

### 4.2. Effects of Prompt Engineering on Triage Performance

To evaluate how prompt engineering impacts triage performance, we observed that no notable improvement in Exp 3 compared to Exp 1 (baseline), with accuracy remaining nearly unchanged at 41.9% (95% CI: 38.8–45.0%) vs. 41.8% (95% CI: 38.7–44.9%). Similar patterns were observed in precision, recall, and F1-score, which indicate that prompt engineering alone may not significantly improve triage performance.

Conversely, Exp 4 achieved an accuracy of 65.5% (95% CI: 62.6–68.4%), which represents a 56.5% improvement over Exp 1 and reduced under-triage to 9.0% (approximately 71.5% reduction). It also improved precision to 68.3% (95% CI: 65.4–71.2%), recall to 65.5% (95% CI: 62.6–68.4%), and F1-score to 0.644 (95% CI: 0.61–0.67). However, it still did not outperform Exp 2 across all metrics.

These findings may be related to the strong reasoning capabilities of advanced LLMs such as GPT-5.2, where zero-shot reasoning without additional instructions can provide robust and reliable results [32]. The applied R-CoT guidance introduced additional structured reasoning guidance and constraints, particularly when synthetic clinical dialogues were included in the input. This additional reasoning structure may have increased the processing burden of integrating information from the dialogues and structured clinical variables, potentially affecting classification performance [33].

This suggests that while CoT reasoning provides measurable improvements, it appears that the inclusion of clinical dialogues may contribute significantly to improving overall performance.

### 4.3. Comparison with Conventional Machine Learning Baselines

To provide a broader reference comparison with conventional supervised machine learning approaches, six machine learning baselines were implemented using the same structured clinical variables employed in Exp 1 (Structured Only). The evaluated models included Random Forest, Gradient Boosting, LightGBM, XGBoost, Logistic Regression, and Decision Tree. All baseline models were trained and evaluated using the same stratified 80/20 train-test split and the same evaluation metrics to ensure a consistent comparison. As shown in Table 9, Random Forest achieved the highest accuracy among the evaluated conventional machine learning baselines (54.5%), followed by Gradient Boosting (51.0%), LightGBM (49.0%), XGBoost (48.5%), Logistic Regression (47.0%), and Decision Tree (36.5%). Compared with Exp 1 (Structured Only), which achieved an accuracy of 41.8%, five of the six evaluated conventional machine learning baselines achieved higher accuracy, with Random Forest obtaining the highest accuracy (54.5%).

### 4.4. Statistical Analysis of Experimental Results

A Cochran’s Q test was performed on the classification results across 1000 cases to assess whether there were performance differences among the four strategies. The results indicated statistically significant differences across the four strategies (p<0.001). Next, post hoc pairwise comparisons were performed using the McNemar test with Bonferroni correction for each pair to identify the source of these differences. The analysis demonstrated that all pairwise comparisons were statistically significant except for the comparison between Exp 1 and Exp 3.

Exp 1 vs. Exp 2, (p<0.001), Difference + 29.30%, (95% CI: 25.15% to 33.45%); Exp 1 vs. Exp 3, (p>0.05), Difference +0.10%, (95% CI: −4.22% to 4.42%); Exp 1 vs. Exp 4, (p<0.001), Difference +23.70%, (95% CI: 19.45% to 27.95%); Exp 2 vs. Exp 3, (p<0.001), Difference −29.20%, (95% CI: −33.35% to −25.05%); Exp 2 vs. Exp 4, (p<0.001), Difference −5.60%, (95% CI: −9.67% to −1.53%); Exp 3 vs. Exp 4, (p<0.001), Difference +23.60%, (95% CI: 19.35% to 27.85%).

These comparisons indicate that both Exp 2 and Exp 4 performed significantly better than the baseline strategies (Exp 1 and Exp 3), which do not incorporate patient dialogues. However, the difference between Exp 2 and Exp 4 was relatively small. Consequently, Exp 2 achieved the best overall performance among all evaluated strategies, while Exp 4 showed the second-best performance.

As observed, these results indicate that incorporating patient dialogues had the most notable impact on improving triage performance, while prompt engineering also led to improvements but to a lesser extent.

To further assess the agreement between the predicted ESI levels and the ground-truth labels, we computed the Weighted Kappa (κ) coefficient for both Exp 1 and Exp 2. Exp 2, which incorporated the generated clinical dialogues, achieved a high level of agreement with the ground-truth labels (κ=0.900). In contrast, Exp 1, which relied solely on structured clinical data without dialogue information, achieved a moderate level of agreement (κ=0.666). These findings provide additional quantitative evidence that the generated patient–nurse dialogues contribute valuable contextual information and improve alignment with the reference ESI classifications.

### 4.5. Clinical Safety and Under-Triage Analysis

From a clinical safety perspective, Exp 2 demonstrated the most significant improvement. Only 22 critical cases were misclassified. The under-triage rate (ESI 1–2 patients) decreased from 68.5% in Exp 1 to 5.5% in Exp 2, which represents a 91.9% relative reduction, as shown in Table 10. This corresponds to a high recall of 94.5% for high-acuity patients (ESI levels 1–2). It should be noted that most errors occurred between adjacent ESI levels associated with lower clinical risk.

These results demonstrate that integrating clinical dialogues may enhance the identification of red-flag symptoms embedded within patient narratives that may not be captured through structured data alone.

**Figure 6:**
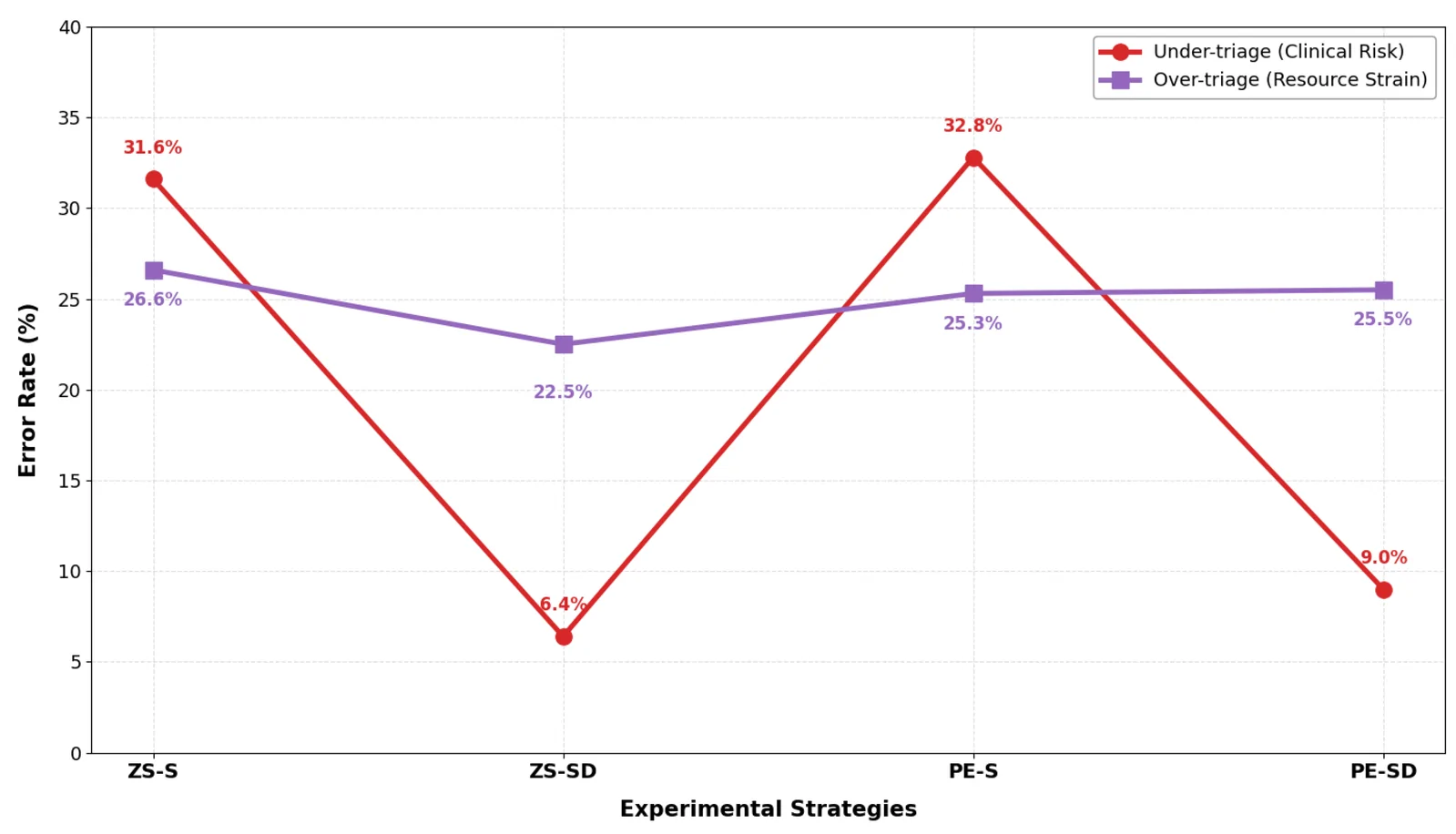
Comparison of clinical safety metrics (under-triage and over-triage rates) across the four experimental configurations. For clarity, the abbreviations represent: ZS-S (Zero-Shot–Structured); ZS-SD (Zero-Shot–Structured & Dialogues); PE-S (Prompt-Engineered–Structured); and PE-SD (Prompt-Engineered–Structured & Dialogues).

Figure 6 presents the clinical safety assessment by comparing under-triage and over-triage rates for all experimental configurations. As shown in the figure, the inclusion of dialogue information is associated with reduced under-triage rates. Over-triage rates remained relatively stable across the experimental configurations. It is notable that Exp 2, which integrated dialogue data, maintained the lowest over-triage rate at 22.5%.

### 4.6. Consistency of Experimental Results

Given the comparisons between Exp 1 and Exp 3 and Exp 2 and Exp 4, the results indicate consistent trends across configurations with dialogue inclusion appearing to contribute more significantly to performance improvements than R-CoT reasoning.

Regarding response times, the inclusion of clinical dialogues slightly increased the model’s response time, as observed in Exp 2 and Exp 4, with an increase of approximately 0.4–2 s. This increase was small relative to the duration of routine ED triage [34,35,36].

#### 4.6.1. ESI-Level Performance Analysis

Table 11 presents a descriptive comparison between Exp 1 and Exp 2 across ESI levels to identify the levels with the greatest improvement. Exp 2 shows notable improvements in classification accuracy, particularly at the most critical levels (ESI 1 and ESI 2). Exp 2 increased accuracy for these levels to 93.0% and 93.5%, respectively, compared with 9.5% and 53.5% in Exp 1.

#### 4.6.2. Confusion Matrix Analysis

**Figure 7:**
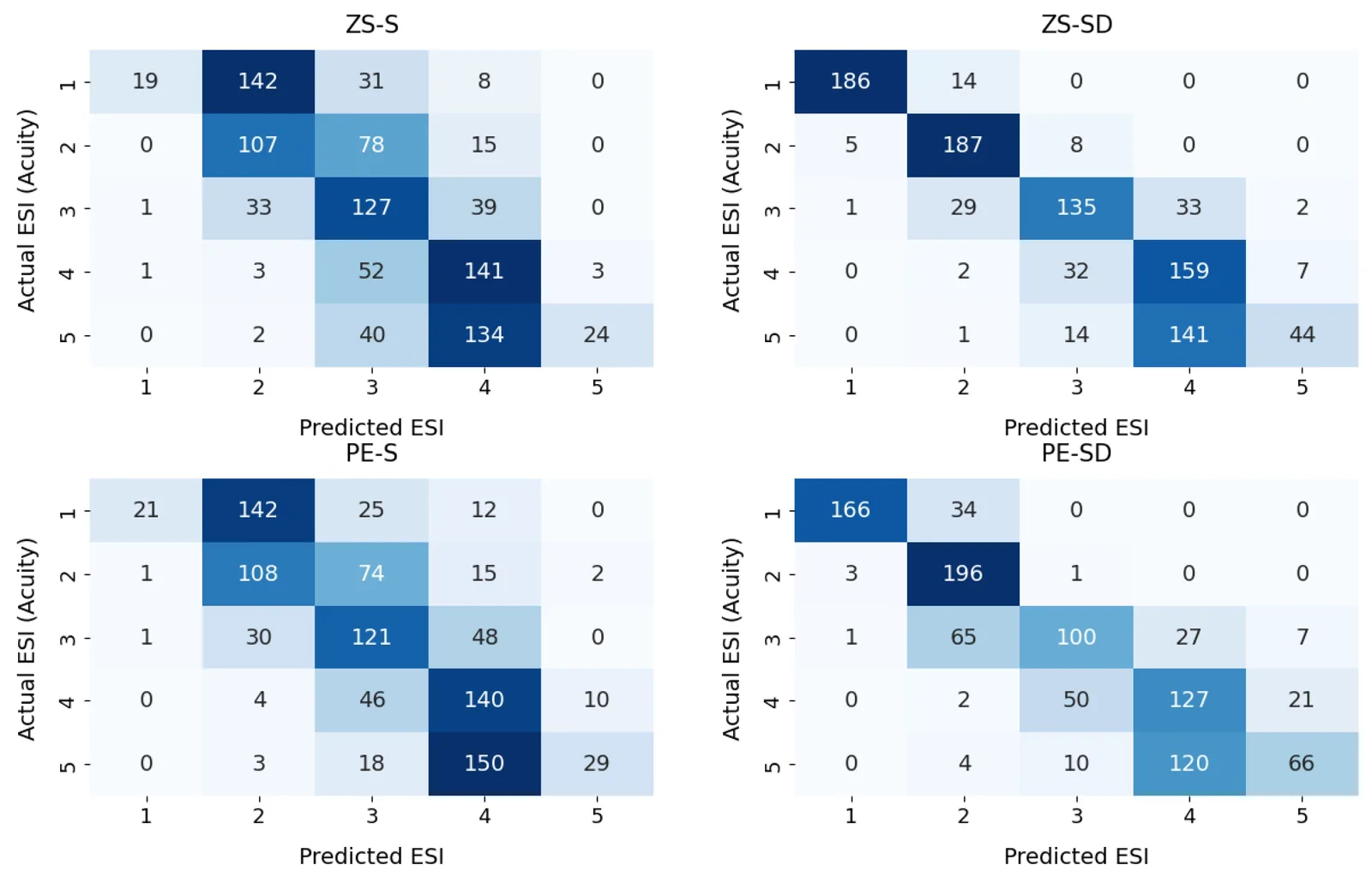
Confusion matrices for the four experimental configurations. For clarity, the abbreviations represent, abbreviations represent: ZS-S (Zero-Shot–Structured); ZS-SD (Zero-Shot–Structured & Dialogues); PE-S (Prompt-Engineered–Structured); and PE-SD (Prompt-Engineered–Structured & Dialogues).

The confusion matrix analysis, shown in Figure 7, indicates improved triage performance when moving from structured-only input to inclusion of patient dialogue. As illustrated, Exp 1 and Exp 3 showed a higher degree of misclassification, with more frequent under-triage errors. In particular in, ESI level 1 cases, where some critical patients were assigned to lower urgency categories.

In contrast, Exp 2 and Exp 4 showed improved diagonal concentration, which suggests improved classification consistency across ESI levels. For instance, the number of correctly identified ESI level 1 cases increased from 19 in Exp 1 to 186 in Exp 2 after integrating dialogue data.

These findings suggest that the inclusion of narrative clinical context not only improves accuracy in critical cases but may also provide complementary information to structured data, potentially contributing to improved identification of clinically relevant patterns and supporting future triage decision-support systems.

### 4.7. Human Evaluation and Inter-Rater Agreement with Emergency Nurses

As Exp 2, which incorporated the generated dialogues, achieved the best overall performance among the four experimental settings, an additional human evaluation was conducted on a randomly selected subset of 50 cases from the full evaluation dataset. To ensure a fair comparison, two experienced emergency nurses independently evaluated the same cases under the same experimental setting as Exp 2, with triage decisions based on both the structured clinical data and the generated dialogues. The nurses assigned ESI levels under blinded conditions, without access to the ground-truth labels or the GPT-5.2 predictions, to minimize potential bias.

The performance of GPT-5.2 and the emergency nurse raters was evaluated against the ground-truth ESI labels using accuracy, Weighted Cohen’s Kappa, under-triage, and over-triage rates, using the same 50 cases and the same sample size for both the model and the nurse raters. As shown in Table 12, GPT-5.2 achieved an accuracy of 72.0% and a Weighted Cohen’s Kappa of 0.902, demonstrating high agreement with the reference ESI classifications. The emergency nurses also demonstrated high agreement with the ground-truth labels, achieving Weighted Cohen’s Kappa values of 0.833 and 0.838 for Nurse 1 and Nurse 2, respectively.

To further assess the consistency of ESI classification among independent evaluators, inter-rater agreement was calculated using Weighted Cohen’s Kappa. The results are presented in Table 13. High agreement was observed across all comparison pairs, including Nurse 1 versus Nurse 2 (κ = 0.842), GPT-5.2 versus Nurse 1 (κ = 0.850), and GPT-5.2 versus Nurse 2 (κ = 0.866).

These findings indicate consistent agreement between GPT-5.2 and experienced emergency nurse raters in assigning ESI levels under the evaluated simulated triage conditions.

## 5. Discussion

This study contributes to the growing body of research on AI-driven emergency triage. While previous ML and DL approaches have shown promising performance using structured clinical variables and textual records [1,3,5,8,11,12,13,14,15], they often rely on structured inputs or simplified textual representations that fail to fully capture the dynamic nature of triage interactions [16]. Even though recent efforts [18] have attempted to process real-world conversations using LLMs, they have largely narrowed their scope to non-critical cases (triage levels 3–5). Notably, such dialogue-only approaches lack access to objective clinical data such as vital signs or physical examination findings, which play a critical role in triage decision-making, and often operate in isolation from structured clinical measurements. Unlike previous works, our study combines clinical dialogues with structured data to provide a more comprehensive representation of the patient’s condition during the triage process. This approach underscores how contextual clinical narratives may refine the interpretation of acuity and facilitate more reliable triage decisions.

The results of the exploratory analysis showed that the generated dialogues primarily provided contextual and narrative enrichment by rephrasing and contextualizing information already available in the structured records, rather than introducing additional clinical facts. This contextual enrichment may help explain the improved triage performance observed when clinical dialogues were integrated with structured data. Accordingly, this study suggests that LLMs may achieve improved triage performance when integrating clinical dialogues with structured clinical data, particularly for high-acuity cases (ESI 1 and 2). The reduction in under-triage observed in Exp 2 indicates that narrative context may play an important role in identifying life-threatening conditions that may not be fully captured by structured vital sign parameters alone. Although Exp 3 and Exp 4, which employed prompt-engineered configurations, showed moderate performance improvements, the application of advanced prompt engineering through R-CoT reasoning did not achieve gains comparable to those observed with clinical dialogues [33]. These findings highlight the relative importance of clinical narrative information compared to prompting strategies.

The additional human evaluation provides complementary evidence supporting the reliability of the proposed dialogue-enhanced triage approach. The high agreement observed between GPT-5.2 and experienced emergency nurses, together with the strong inter-rater agreement among the nurse raters, suggests that the model’s ESI classifications are consistent with expert clinical judgment under the evaluated simulated triage conditions. These findings complement the quantitative improvements observed in Exp 2 and further support the value of incorporating generated dialogues into the triage process.

To further contextualize these findings, multiple conventional supervised machine learning baselines were evaluated using the same structured clinical variables employed in the structured-only configurations (Exp 1 and Exp 3). The evaluated baselines included Random Forest, Gradient Boosting, LightGBM, XGBoost, Logistic Regression, and Decision Tree. Among these models, Random Forest achieved the highest accuracy (54.5%), while the structured-only LLM configurations achieved 41.8% and 41.9% accuracy in Exp 1 and Exp 3, respectively. The Structured & Dialogues configurations achieved higher accuracies of 71.1% and 65.5% in Exp 2 and Exp 4, respectively. Overall, the structured-data-only machine learning baselines achieved higher accuracy than the structured-only LLM configurations, while the dialogue-enhanced LLM configurations showed higher accuracy than their corresponding structured-only configurations.

### 5.1. Clinical Safety and Triage Performance

From a clinical safety perspective, reducing under-triage is more critical than maximizing overall accuracy alone, as patient safety primarily depends on the correct identification of high-risk cases [37,38]. The reduction in under-triage associated with dialogue inclusion indicates that LLM-assisted triage systems may have the potential to support safer decision-making in emergency scenarios, particularly in high-demand mass-gathering environments. Although under-triage is associated with greater clinical risk, controlling over-triage remains essential to avoid unnecessary emergency department resource utilization. The findings show that incorporating dialogue improves sensitivity to critical cases. Importantly, this improvement is achieved without significantly increasing over-triage, suggesting potential implications for patient safety while avoiding excessive over-triage and associated resource utilization.

Among all configurations, Exp 2 demonstrated the most balanced performance, particularly in maintaining lower over-triage rates compared to other configurations. Although the incorporation of R-CoT reasoning in subsequent experiments provided only marginal improvements in selected metrics, the results suggest that the inclusion of clinical dialogues provides richer contextual information that may support more accurate triage decisions without introducing excessive conservative bias [39]. This finding suggests that contextual richness may play an important role in improving model reliability, enabling more consistent triage behavior while limiting both under-triage and over-triage.

### 5.2. Response Time and Operational Efficiency

As previously discussed, the slight increase in response time associated with dialogue inclusion or the application of prompt engineering was small relative to the duration of routine ED triage (approximately 0.4–2 s) [34,35,36]. This level of efficiency is particularly relevant in mass-gathering environments, where timely decision-making is important due to high patient volumes and the need for efficient triage support.

Given the observed improvements in performance, particularly in the early identification of high-acuity ESI 1–2 cases, the additional computational overhead appears to be operationally manageable in time-sensitive emergency settings. This balance between response latency and triage accuracy suggests that the proposed system may have the potential to be applicable in real-world high-pressure clinical environments, subject to further validation in practical deployment scenarios.

### 5.3. Ethical Considerations and Trustworthy AI Deployment

The deployment of LLM-based triage systems such as GPT-5.2 in high-pressure healthcare environments requires careful consideration of ethical and societal factors. A key concern is the potential for algorithmic bias, which may affect fairness in patient care. In this study, a pre-processing strategy based on a balanced and stratified dataset was adopted to reduce potential demographic imbalance [39]. The dataset was constructed with equal representation across genders (500 males and 500 females) and across all ESI acuity levels within the 1000-case subset, which supports more uniform model evaluation across patient groups. In addition, accountability is ensured through a human-in-the-loop validation framework. This ensures that the system operates as a clinical decision-support tool, while final decisions remain under clinician supervision. This design helps reduce the risk of unintended or misinterpreted model outputs and supports clinical safety in deployment scenarios. Transparency is further supported through detailed reporting of evaluation metrics and study limitations. Hence, these considerations contribute to the development of responsible AI systems and support their safe use in complex healthcare environments. Ensuring fairness, accountability, and transparency remains important for maintaining clinical integrity and supporting equitable care delivery across patient populations [38,39].

### 5.4. Implications for AI-Driven Triage Systems

The findings of this study provide several important implications for the design and evolution of AI-assisted triage systems. First, the superior performance of Exp 2, which integrated clinical dialogues, suggest a potential shift from data-centric toward context-centric AI approaches in emergency medicine. Notably, its outperformance relative to configurations incorporating R-CoT indicates that, in rich clinical dialogues, the model’s inherent ability to process unstructured narrative information is sufficiently effective without additional reasoning constraints. In contrast, R-CoT structured reasoning steps that may limit the model’s ability to fully exploit rich clinical narratives compared to the more direct approach used in Exp 2 [33].

Furthermore, the results suggest that zero-shot LLM configurations can leverage unstructured dialogue data without requiring task-specific fine-tuning. This indicates that contextual richness may be more important than increased prompting complexity, as narrative inputs can provide additional clinical information that is not always fully captured in structured data. Overall, these findings suggest the potential for developing context-aware decision-support systems for use in dynamic, high-pressure emergency care environments, with potential implications for supporting patient safety and clinical efficiency [38,39].

### 5.5. Clinical Implications and Practical Applicability

This study highlights the potential value of integrating clinical dialogues with structured data within high-pressure mass-gathering triage workflows in Hajj-context simulated settings. These findings support the proposed multimodal framework in which structured clinical data and narrative dialogue are jointly utilized to enhance triage decision support and improve the identification of high-risk clinical cases. Furthermore, this approach may facilitate integration into existing triage workflows without disrupting routine clinical operations. This may support its suitability for large-scale, resource-constrained environments where efficiency and minimal intervention are critical. Importantly, the proposed approach is intended to augment rather than replace clinical decision-making, with human oversight remaining essential for ensuring patient safety and diagnostic reliability [38].

### 5.6. Research Limitations and Future Research Directions

Despite its achievements, there are several limitations of this study that future research should address. A primary limitation is that the dialogues were generated using GPT-4.1 based on structured clinical data and the corresponding ground-truth ESI labels, rather than being derived from real nurse–patient interactions. Consequently, the generated dialogues may not fully capture the complexity, variability, and contextual nuances of real-world clinical communication, which may affect the generalizability of the reported results. It is also worth noting that this work relies on nurse-assigned triage labels as the ground truth. Although an additional human evaluation demonstrated high inter-rater agreement among experienced emergency nurses, this evaluation was conducted on a subset of the dataset under simulated conditions. Therefore, further validation involving larger cohorts of emergency nurse raters across multiple clinical settings is warranted to further assess the generalizability and robustness of the proposed dialogue-enhanced triage approach. Another limitation is the lack of real-world validation. The current simulation-based evaluation does not fully capture the inherent complexities and operational constraints of clinical workflows. Although the proposed framework may offer potential practical advantages, the reliance on synthetic dialogues rather than a fully deployed real-time system could limit the generalizability of the findings. Additionally, the framework was evaluated only in a text-based controlled simulation setting, without any voice-based interaction or human–AI collaborative evaluation. Consequently, future work should conduct end-to-end validation to assess performance under actual emergency triage conditions. Additionally, this study assessed the model’s performance using clear and unambiguous Arabic inputs, which does not fully reflect the linguistic diversity, regional accents, and multilingual communication patterns (e.g., Urdu, Indonesian, and English) commonly encountered among international pilgrims. Moreover, the evaluation did not include voice-based (spoken) conversations, audio interference, or background noise, all of which are unavoidable in densely crowded Hajj environments. As a result, the reported findings may have limited generalizability to the complex acoustic and linguistic conditions present in real-world mass gathering scenarios. Therefore, future studies should conduct evaluations in multilingual and noisy environments. In addition, this study was conducted using a single LLM (GPT-5.2). Although multiple conventional supervised machine learning baselines, including Random Forest, Gradient Boosting, LightGBM, XGBoost, Logistic Regression, and Decision Tree, were evaluated using the same structured clinical variables, no benchmarking against other state-of-the-art LLMs was performed. The primary objective of this study was to conduct a controlled evaluation to isolate the effect of clinical dialogues on ESI triage performance under consistent experimental conditions rather than to compare multiple LLM architectures. Therefore, the results primarily focus on intra-model analysis and comparison with conventional structured-data-only baselines. Future work should include benchmarking against state-of-the-art LLMs to provide comprehensive cross-model comparisons and further evaluate the generalizability of the proposed approach. Finally, the absence of non-verbal clinical cues in the model’s evaluation is another concern. In clinical practice, experienced triage nurses heavily depend on critical visual and auditory indicators such as a patient’s gait, facial expressions of pain, and overall physical appearance to assess patient urgency [30]. The exclusion of these indicators may affect the accuracy of triage assessment. Accordingly, future work should investigate the inclusion of such indicators to capture the full clinical context required to assess patient severity, which may go beyond what can be conveyed by numerical or textual data alone. However, addressing these operational and technical challenges is important to support the potential deployment of AI-driven triage systems in large-scale emergency environments.

## 6. Conclusions

Our findings demonstrate that integrating clinical dialogues with structured data can improve LLM performance in emergency triage. In particular, the Zero-Shot (Structured & Dialogues) configuration achieved the highest accuracy and the lowest under-triage error rates. This suggests that contextual patient narratives may have a greater impact on performance than prompt engineering alone. Collectively, these results highlight the potential value of combining structured physiological data with patient narratives to improve triage reliability and decision quality.

This study also presents a conceptual framework introducing a dual-layer feedback mechanism designed to support patient safety and guide future improvements in high-demand emergency settings.

While these findings are promising, they should be interpreted within the context of a controlled simulation-based evaluation using synthetically generated clinical dialogues. Further validation using real-world clinical data and in actual clinical settings remains necessary.

## Figures and Tables

**Table 1:** Overview of databases used.

No.	Database	Extracted Variables
1	MIMIC-IV-ED v2.2	Temperature, Heart Rate, Respiratory Rate, Oxygen Saturation, Systolic and Diastolic Blood Pressure, Pain Score, Acuity Level, Chief Complaint, Patient Visit Identifiers (subject_id, stay_id).
2	MIMIC-IV v3.1	Age, gender, patient visit identifier (subject_id).

**Table 2:** Clinical variables extracted from the MIMIC-IV-ED and MIMIC-IV databases.

Variable Name	Variable Description	Type
subject_id	Unique anonymized identifier for each patient.	Identifier
stay_id	Unique anonymized identifier for each ED visit.	Identifier
anchor_age	Patient age at the time of the visit (years).	Numerical
gender	Patient biological sex (Male or Female).	Categorical
temperature	Body temperature (°C).	Numerical
heartrate	Heart rate (beats per minute, bpm).	Numerical
resprate	Respiratory rate (breaths per minute).	Numerical
o2sat	Oxygen saturation level (${\mathrm{SpO}}_{2}$%).	Numerical
sbp	Systolic blood pressure (mmHg).	Numerical
dbp	Diastolic blood pressure (mmHg).	Numerical
pain	Patient-reported pain score (0–10 scale).	Numerical
acuity	ESI triage level (Levels 1–5).	Categorical
chiefcomplaint	Patient’s primary reported complaint at triage	Text

**Table 3:** Gender distribution.

Gender	Percentage
Female	54.10%
Male	45.90%

**Table 4:** Age groups.

Age Group	Percentage
Adult	73.64%
Elderly	25.33%
Child	1.03%

**Table 5:** ESI acuity level.

Acuity	Percentage
ESI 3	52.96%
ESI 2	32.80%
ESI 4	6.71%
ESI 1	5.65%
ESI 5	0.26%

**Table 6:** Health conditions included in the Hajj context adaptation.

Health Category	Included Conditions
Metabolic and Endocrine	diabetes mellitus, hyperglycemia, hypoglycemia, insulin-related conditions, electrolyte imbalance.
Gastrointestinal	foodborne illness, vomiting, diarrhea, dehydration.
Cardiovascular	cardiovascular conditions, chest pain, cardiac-related symptoms.
Musculoskeletal and Trauma	ankle injuries, sprains, joint pain, muscle pain, fatigue, strain, trauma, falls, fractures, post-surgical conditions.
Respiratory	respiratory symptoms, shortness of breath, cough.
Neurological and Other Systems	neurological conditions, altered mental status, non-specific pain, gastrointestinal disorders.
Accidents and External Causes	accidents, road traffic accidents (RTA), assault-related injuries.
Other Specialties	fever, rheumatological conditions, orthopedic conditions, ophthalmological conditions, dermatological conditions, ENT conditions, psychiatric conditions, oncological conditions, genitourinary conditions, dental conditions, other non-specific conditions.

**Table 7:** Summary of experimental strategies, input data configurations, and prompting techniques used in this study.

Exp#	Strategy Name	Input Data	Prompting Technique
Exp 1	Zero-Shot–Structured Only (Baseline)	Structured clinical data only	Standard prompt
Exp 2	Zero-Shot–Structured & Dialogues	Structured clinical data and clinical triage dialogues	Standard prompt
Exp 3	Prompt-Engineered–Structured Only (Baseline)	Structured clinical data only	Rule-constrained Chain-of-Thought (R-CoT)
Exp 4	Prompt-Engineered–Structured & Dialogues	Structured clinical data and clinical triage dialogues	R-CoT

**Table 8:** Performance metrics across experiments.

Metrics	Exp 1	Exp 2	Exp 3	Exp 4
**Accuracy (%)**	41.8 95% CI: (38.7–44.9)	**71.1** **95% CI: (68.3–73.9)**	41.9 95% CI: (38.8–45.0)	65.5 95% CI: (62.6–68.4)
**Precision (%)**	59.4 95% CI: (56.4–62.5)	**75.9** **95% CI: (73.3–78.5)**	56.1 95% CI: (53.0–59.2)	68.3 95% CI: (65.4–71.2)
**Recall (%)**	41.8 95% CI: (38.7–44.9)	**71.1** **95% CI: (68.3–73.9)**	41.9 95% CI: (38.8–45.0)	65.5 95% CI: (62.6–68.4)
**F1-Score**	0.366 95% CI: (0.34–0.40)	**0.690** **95% CI: (0.66–0.72)**	0.374 95% CI: (0.34–0.40)	0.644 95% CI: (0.61–0.67)
**Response Time (s)**	2.56	**2.99**	4.56	4.72
**Under-triage (%)**	31.6	**6.4**	32.8	9.0
**Over-triage (%)**	26.6	**22.5**	25.3	25.5

Exp 1: Zero-Shot–Structured Only (Baseline); Exp 2: Zero-Shot–Structured & Dialogues; Exp 3: Prompt-Engineered–Structured Only (Baseline); Exp 4: Prompt-Engineered–Structured & Dialogues; CI denotes the 95% Confidence Interval.

**Table 9:** Performance of conventional machine learning baselines using structured clinical variables.

Model	Accuracy (%)	Precision (%)	Recall (%)	F1-Score (%)	Under-Triage (%)	Over-Triage (%)
**Random Forest**	**54.50**	**54.80**	**54.50**	**54.60**	**26.50**	**19.00**
Gradient Boosting	51.00	51.60	51.00	51.20	28.00	21.00
LightGBM	49.00	50.00	49.00	49.30	30.50	20.50
XGBoost	48.50	48.80	48.50	48.60	27.00	24.50
Logistic Regression	47.00	47.20	47.00	46.90	29.50	23.50
Decision Tree	36.50	36.90	36.50	36.50	32.00	31.50

**Table 10:** Comparative analysis of clinical safety and under-triage rates for critical cases (ESI 1 and 2) between Exp 1 and Exp 2.

Experiment	Total Cases	Under-Triage	Under-Triage	High-Acuity Recall
	**(ESI 1–2)**	**Cases**	**Rate (%)**	**(%)**
Exp 1—Structured Only (Baseline)	400	274	68.5	31.5
**Exp 2—Structured & Dialogues**	**400**	**22**	**5.5**	**94.5**

ESI 1 and 2: Levels 1–2 represent high-acuity or life-threatening cases. Under-Triage Rate: Percentage of critical cases (ESI 1–2) incorrectly assigned to a lower clinical priority. High-Acuity Recall: Percentage of high-acuity cases (ESI 1–2) correctly identified as high-acuity.

**Table 11:** Comparative accuracy analysis for each ESI Level between Exp 1 and Exp 2.

Experiment	Level 1	Level 2	Level 3	Level 4	Level 5	Overall Accuracy (%)
Exp 1: Structured Only (Baseline)	9.50	53.50	63.50	70.50	12.00	**41.80**
**Exp 2: Structured & Dialogues**	**93.00**	**93.50**	**67.50**	**79.50**	**22.00**	**71.10**

**Table 12:** Performance of GPT-5.2 and emergency nurse raters against the ground truth.

Rater	Accuracy (%)	Weighted Cohen’s Kappa	Under-Triage (%)	Over-Triage (%)
Nurse 1	56.00	0.833	18.00	26.00
Nurse 2	70.00	0.838	8.00	22.00
**GPT-5.2 (Model)**	**72.00**	**0.902**	**8.00**	**20.00**

**Table 13:** Inter-rater agreement in ESI classification.

Comparison Pair	Weighted Cohen’s Kappa	Interpretation
Nurse 1 vs. Nurse 2	0.842	Almost perfect agreement
GPT-5.2 vs. Nurse 1	0.850	Almost perfect agreement
GPT-5.2 vs. Nurse 2	0.866	Almost perfect agreement

## Data Availability

The clinical data used in this study are available from the MIMIC-IV and MIMIC-IV-ED databases. These datasets are publicly accessible upon completion of the required training, including the Health Insurance Portability and Accountability Act (HIPAA) certification and the CITI Program for human subjects research. Access can be requested through the official PhysioNet platform at: https://physionet.org/. The structured clinical data used in this study are subject to the MIMIC data use agreement and therefore cannot be redistributed by the authors. However, all prompts, model configurations, and dialogue generation procedures are provided in the Supplementary Materials to ensure reproducibility.

## Supplementary Materials

The following supporting information can be downloaded at: https://www.mdpi.com/article/10.3390/diagnostics16203268/s1, The Supplementary Materials include: (1) the full prompt used for synthetic clinical dialogue generation, (2) the ESI classification prompts across all experimental configurations, (3) post-processing and error handling procedures, (4) a representative synthetic dialogue example, (5) a case-level prediction example, and (6) model and experimental configuration.

## Abbreviations

The following abbreviations are used in this manuscript:

ED	Emergency Department
ESI	Emergency Severity Index
AI	Artificial Intelligence
ML	Machine Learning
DL	Deep Learning
LLM	Large Language Model
EHR	Electronic Health Record
STT	Speech-to-Text
R-CoT	Rule-constrained Chain of Thought
