# Supplementary material for: Evaluating a Large Language Model for Emergency Triage Using Synthetic Clinical Dialogues: Toward a Multimodal Clinical Safety Framework
Source: Diagnostics (Basel). 2026 Oct 9;16(20):3268. doi: 10.3390/diagnostics16203268 (PMC13703718; doi:10.3390/diagnostics16203268)
Supplement: Supplementary file 1 [file diagnostics-16-03268-s001.zip › diagnostics-4496179-supplementary.pdf]

LLM-Based Reproducibility and Experimental Pipeline Details

1. Synthetic Clinical Dialogue Generation Prompt

The following prompt was used to generate synthetic nurse–patient clinical dialogues based on structured clinical inputs:

You are an expert Medical Simulation Engineer specializing in Emergency Department (ED) triage. Your task is to convert structured clinical data into a short, realistic triage dialogue in Arabic between a triage nurse and a Hajj pilgrim. The dialogue must reflect real-world emergency triage practice and strictly adhere to the Emergency Severity Index (ESI), Version 4.

CRITICAL MANDATORY RULE:

You are an expert medical simulation Engine specializing in Emergency Department triage. Your task is to generate a short, realistic emergency triage dialogue in Arabic between a triage nurse and a Hajj pilgrim based on structured clinical data.

INPUT DATA:

-Age
-Gender
-Vital signs (HR, BP, RR, Temp, O2Sat)
-Chief complaint
-Pain score
-Assigned ESI level (ground truth)

HAJJ CONTEXT:

Anchor all symptoms in realistic Hajj-related scenarios (e.g., Tawaf, Sa'i, Jamarat, walking long distances, heat exposure, crowd density, exhaustion).

CRITICAL RULE:

The assigned ESI level (ground truth) MUST guide dialogue realism.

If ESI 1 or 2 is assigned, simulate appropriate severity even if vital signs appear stable (e.g., sudden collapse, severe distress, or neurological impairment).

CLINICAL REALISM:

-Do NOT explicitly mention ESI level, diagnosis, or medical management.
-The acuity must be implied through behavior and dialogue only.
-Dialogue must start immediately without greetings.
-Maximum 3–5 turns (shorter for ESI 1–2).

LANGUAGE:

-Arabic only
-Natural patient expressions (no medical jargon)

ESI BEHAVIOR SIMULATION:

ESI 1: unconscious or minimally responsive
ESI 2: severe distress, short responses
ESI 3: stable but symptomatic
ESI 4: mild symptoms, limited need
ESI 5: stable, reassurance-seeking

RESOURCE LOGIC (INTERNAL ONLY):

Used only to guide realism (NOT mentioned in output):
Resources include:
-Laboratory tests
-Imaging (X-ray, CT, MRI)
-IV fluids
-IV/IM medications
-Specialist consultation
Not resources:
-Physical examination
-Oral medications
-Prescriptions
-Tetanus vaccination
-Simple wound care

OUTPUT FORMAT:

Nurse :
Patient:

Note: The chief complaint is provided as part of the structured input and is jointly processed with other clinical variables (e.g., vital signs, pain score, and ESI level) within the generation prompt. This enables the language model to generate a coherent and clinically grounded simulated Hajj-context triage scenario, where symptom presentation and severity are consistently aligned with the corresponding ESI category.

2. ESI Classification Prompts

The following prompts were used for Emergency Severity Index (ESI) classification across four experimental configurations:

1. Exp1 Prompt: Zero-Shot – Structured Clinical Data Only (Baseline):

```
You are an expert emergency triage nurse.

Based on the following structured clinical vitals, assign an Emergency Severity Index (ESI) level from 1 to 5.

----------------------------

OUTPUT FORMAT (JSON ONLY):

----------------------------

{
  "ESI_level": 1-5,
  "justification": "Short clinical rationale (1–2 sentences)"
}
```

2. Exp2 Prompt: Zero-Shot – Structured Clinical Data + Clinical Dialogues:

```
You are an expert emergency triage nurse.

Based on the following structured clinical vitals AND the provided triage dialogue, assign an Emergency Severity Index (ESI) level from 1 to 5.

----------------------------

OUTPUT FORMAT (JSON ONLY):

----------------------------

{
  "ESI_level": 1-5,
  "justification": "Short clinical rationale combining structured clinical data and dialogue context (1–2 sentences)"
}
```

3. Exp3 Prompt: Rule-Constrained Chain-of-Thought (R-CoT) – Structured Clinical Data Only (Baseline):

```
You are an expert Emergency Triage Nurse with 20 years of experience, operating under
the Emergency Severity Index (ESI), Version 4. Your task is to assign an ESI level (1–5)
based only on structured clinical data.

----------------------------

RESOURCE ESTIMATION RULES:

----------------------------

- Non-resource (0): physical examination, oral medications, prescriptions, tetanus
immunization, simple wound dressing.
- Resource-based: laboratory tests, imaging (X-ray/CT/MRI), IV fluids, IV/IM
medications, specialty consultation.
----------------------------

TRIAGE DECISION RULES:

----------------------------

1. Life-threatening condition requiring immediate intervention → ESI 1
2. High-risk situation or severe pain requiring immediate attention → ESI 2
   (Pain alone does not upgrade ESI unless clinically justified)
3. Otherwise, based on resource utilization:
   - ≥2 resources → ESI 3
   - 1 resource → ESI 4
   - 0 resources → ESI 5
----------------------------

REASONING STEPS (R-CoT):

----------------------------

1. Does the patient require a life-saving intervention? (Yes/No)
2. Is the patient high-risk? (Yes/No)
3. Estimate required resources (list and count)
4. Explain how resources influence ESI decision
5. Provide final reasoning in 2–3 sentences based on ESI guidelines
----------------------------

OUTPUT FORMAT (JSON ONLY):

----------------------------

{
  "ESI_level": 1-5,
  "Predicted_resources": {
    "resources_list": [],
    "resources_count": 0,
```

```
  "justification": {
    "life_saving_intervention": "Yes/No",
    "high_risk_situation": "Yes/No",
    "final_reasoning": "2–3 sentences"
  }
 }
}
```

4. Exp4 Prompt: Rule-Constrained Chain-of-Thought (R-CoT) – Structured Clinical Data + Clinical Dialogues:

```
You are an expert Emergency Triage Nurse with 20 years of experience, operating under
the Emergency Severity Index (ESI), Version 4. Your task is to assign an ESI level (1–5)
based on structured clinical data and clinical dialogue.

----------------------------

RESOURCE ESTIMATION RULES:

----------------------------

- Not counted as a resource (0): physical examination, oral medications, prescriptions,
tetanus immunization, simple wound dressing.
- Counted as a resource: laboratory tests (blood/urine), imaging (X-ray, CT, MRI), IV
fluids, IV/IM medications, specialty consultation.
----------------------------

TRIAGE DECISION LOGIC:

----------------------------

1. Life-threatening condition → ESI 1
2. High-risk condition or severe pain requiring immediate intervention → ESI 2
  (Pain alone does not upgrade ESI unless clinically justified)
3. Otherwise based on resource utilization:
  - ≥2 resources → ESI 3
  - 1 resource → ESI 4
  - 0 resources → ESI 5
----------------------------

CLINICAL PRIORITIZATION RULES:

----------------------------

- Prioritize abnormal vital signs over patient narrative.
- Extract critical red-flag symptoms from dialogue (e.g., chest pain, syncope, severe
dizziness, confusion, inability to walk).
- Use dialogue to detect silent or hidden risks not reflected in vital signs.
- In borderline cases, dialogue information should be used to prevent under-triage.
----------------------------
```

REASONING STEPS (R-CoT):

----

1. Life-saving intervention required? (Yes/No)
2. High-risk situation present? (Yes/No)
3. Estimate required resources (list and count)
4. Explain contribution of each resource to decision
5. Final reasoning (2–3 sentences combining vitals + dialogue)

OUTPUT FORMAT (JSON ONLY):

```
{
  "ESI_level": 1-5,
  "Predicted_resources": {
    "resources_list": [],
    "resources_count": 0,
    "justification": {
      "life_saving_intervention": "Yes/No",
      "high_risk_situation": "Yes/No",
      "final_reasoning": "2–3 sentences"
    }
  }
}
```

3. Post-Processing and Error Handling:

To ensure output consistency and data quality, a post-processing procedure was applied to all model outputs. Responses that did not conform to the required structured format (e.g., non-JSON outputs, missing fields, or invalid ESI labels outside the range of 1–5) were identified during output inspection. This procedure ensured that all outputs satisfied the predefined formatting and labeling requirements before inclusion in the final analysis.

4. Representative Synthetic Dialogue Example

The following is an example of a simulated nurse–patient dialogue corresponding to ESI level 3.

Input Patient Clinical Data:

-Demographics: Female, 20 years old
-Chief Complaint: Abdominal pain
-Pain Score: 5/10
-Blood Pressure (BP): 124/80 mmHg
-Oxygen Saturation (O_2Sat): 98% on room air
-Respiratory Rate (RR): 16 breaths per minute
-Heart Rate (HR): 90 beats per minute
-Temperature: 37°C (98.6°F)
-Assigned ESI Level: 3

Generated Dialogue (Original Arabic) :

Nurse: أين بالضبط تشعرين بالألم في بطنك؟ ومتى بدأ هذا الألم؟
Patient: بدأ الألم منذ حوالي ساعتين، في الجهة اليمنى السفلية من البطن، ويزداد عند المشي أو الحركة.
Nurse: هل شعرتِ بالغثيان أو القيء؟ أو أي ارتفاع في درجة الحرارة أو مشاكل في التبول؟
Patient: نعم، أشعر بالغثيان بشكل خفيف، لكن لم يحدث قيء. □ توجد حرارة أو مشاكل في التبول.
Nurse: هل تمكنتِ من إكمال الطواف والسعي أم اضطررتِ للتوقف؟
Patient: حاولت الإكمال لكن اضطررت للتوقف والجلوس بسبب شدة الألم.

English Translation:
Nurse: Where exactly do you feel the pain in your abdomen, and when did it start?
Patient: The pain started about two hours ago in the lower right side of my abdomen, and it gets worse when I walk or move.
Nurse: Have you experienced nausea or vomiting, any fever, or any problems with urination?
Patient: Yes, I have mild nausea, but I have not vomited. I do not have a fever or any problems with urination.
Nurse: Were you able to complete the Tawaf and Sa'i, or did you have to stop?
Patient: I tried to continue, but I had to stop and sit down because of the severity of the pain.

5. Case-Level Prediction Example (Exp2: Structured Clinical Data + Clinical Dialogue)

Case ID: 001 (De-identified)

Input Patient Clinical Data:

-Demographics: Male, 54 years old
-Chief Complaint: Chest pain
-Pain Score: 8/10
-Blood Pressure (BP): 167/84 mmHg
-Oxygen Saturation (O_2Sat): 100% on room air
-Respiratory Rate (RR): 24 breaths per minute
-Heart Rate (HR): 92 beats per minute
-Temperature: (97°F)

Clinical Dialogue (Generated in Arabic):

Nurse: هل تشعر بألم شديد في صدرك الآن؟
Patient: نعم، الألم قوي جداً ويزيد كلما أتنفس.
Nurse: هل تشعر بضيق في التنفس أو دوخة؟
Patient: أشعر بدوخة شديدة وكأن الأرض تدور بي، و□ أستطيع الوقوف جيداً بعد الطواف.
Nurse: هل سبق أن شعرت بهذا الألم من قبل؟
Patient: أبداً، أول مرة يحصل معي هكذا وأنا خائف.

English Translation:
Nurse: Are you experiencing severe chest pain right now?
Patient: Yes, the pain is very severe and gets worse when I breathe.
Nurse: Are you experiencing shortness of breath or dizziness?
Patient: I feel very dizzy, as if the ground is spinning around me, and I cannot stand properly after completing Tawaf.
Nurse: Have you experienced this pain before?
Patient: Never. This is the first time I have experienced anything like this, and I am scared.

Model Output (Exp2):

ESI Level: 2

54M with new-onset severe chest pain (pain 8/10) worse with breathing plus significant dizziness/near-syncope and tachypnea (RR 24). Although oxygenation is normal and no hypotension, symptoms suggest a high-risk condition (e.g., ACS/PE) requiring immediate evaluation and rapid diagnostics, but not currently requiring life-saving intervention.

6– Model and Experimental Configuration

This section summarizes the model configurations used for both synthetic dialogue generation and ESI classification experiments.

Models

- Synthetic Dialogue Generation: GPT-4.1 (API version specified in the implementation environment).
- ESI Classification: GPT-5.2 (API version specified in the implementation environment).

Generation Settings (GPT-4.1)

- Temperature: 0.7
- Top-p: Default (not modified)
- Seed: Not explicitly set (default system behavior)
- Max tokens: Default API configuration.

Classification Settings (GPT-5.2)

- Temperature: 0.0 (deterministic setting) .
- Top-p: Default (not modified).
- Seed: Not explicitly set (default system behavior).
- Max tokens: Default API configuration.

Purpose

These configurations were used to ensure:

- Controlled and reproducible synthetic dialogue generation.
- Stable and deterministic ESI classification.
- Fair comparison across all experimental settings.

Notes

No model fine-tuning or parameter optimization was performed. All variations in outputs are solely due to input conditions defined in the experimental design.
